# Long-term trends in urban-neighbourhood inequalities in cause-specific mortality and hospitalisation – multilevel analyses among individuals nested in Finnish post-code areas, 1991–2018

**DOI:** 10.1016/j.ssmph.2022.101323

**Published:** 2022-12-17

**Authors:** Lasse Tarkiainen, Pekka Martikainen

**Affiliations:** a University of Helsinki, Population Research Unit, URBARIA Helsinki Institute of Urban and Regional Studies, Unioninkatu 35, 00014, Helsingin Yliopisto, Finland; b University of Helsinki, Population Research Unit, Helsinki, Finland; c Max-Planck-Institute for Demographic Research, Rostock, Germany

**Keywords:** Mortality, Inequality, Neighborhoods, Trend, Hospitalisation

## Abstract

**Background:**

High-income countries yield mixed evidence concerning the long-term trends of neighbourhood inequalities in health outcomes. The reasons why these inequalities persist and the factors driving any changes over time remain unclear. We analysed trends in general neighbourhood differences in mortality and hospitalisation, compared specific area-level and individual-level income effects, and assessed whether area-level effects were attributable to the neighbourhood population composition.

**Methods:**

This prospective cohort study used individual-level register-linked information on sociodemographic factors covering the total population of 20–64-year-olds living in Finnish cities at the beginning of seven four-year periods in 1991–2018 (N = 952,493–1,200,431). We used random-effects Poisson models to assess all-cause and external mortality and hospitalisations among individuals nested in postal-code areas.

**Results:**

The general contextual effect of the neighbourhood on all-cause mortality and hospitalisation was stable across time, with a median incidence-rate ratio of around 1.20–1.30, and it was mainly attributable to the population's composition. The association between area-level income and both mortality and hospitalisation was also robust and increased slightly even after accounting for population composition. The lowest neighbourhood income quintile in 2015–2018 had 15% (95% CI:5–26%) and 30% (95% CI:15–47%) excess mortality among men and women, respectively. These differentials were particularly large for external causes, but all area-level income associations were much smaller than the corresponding individual-level associations.

**Conclusion:**

The overall relevance of the neighbourhood context to mortality and hospitalisation was stable across time, and generally attributable to population composition. However, there were substantial relative area-level income disparities between neighbourhoods, which had grown over time.

## Highlights

• No studies have explored trends in the general contextual effects of neighbourhoods on mortality.
• The relevance of the neighbourhood context was stable across time and largely attributable to the population composition.
• Area-level income disparities in mortality were substantial and had mainly grown over time.
• Most of the processes driving health inequalities in Finland operate on other than the neighbourhood level.

## 1 Introduction

Numerous studies conducted in high-income countries have reported that health and mortality are unevenly distributed not only between socioeconomic groups but also among urban residential neighbourhoods. Individuals with a low socioeconomic position have high incidence of health problems and mortality, and the pattern is similar on the area level as high mortality and morbidity cluster in more deprived neighbourhoods (Diez Roux & Mair, 2010). It is suggested that the mechanisms behind the association between socioeconomic position and health operate mainly on the individual level, and that area-level disparities reflect neighbourhood differences in population composition, although area-level processes may also affect the health of residents independently of individual characteristics. For example, the health of residents in certain urban neighbourhood may suffer from insufficient local health services, exposure to environmental health risks (e.g. air pollution), heavy traffic, lack of green spaces, and high risk of violent crime. Local social processes and norms may also promote behaviour that is hazardous to health and wellbeing (Diez Roux & Mair, 2010; Galster et al., 2012). These adverse neighbourhood characteristics often co-occur with low socioeconomic position at the individual level. This complicates identifying and studying the mechanisms behind the possible independent effect of the neighbourhood socioeconomic characteristics given the strong inverse association between individual level socioeconomic position and health.

The relevance of the residential area to individuals’ health and well-being may be interpreted in various ways. Merlo et al. (Merlo et al., 2018) suggest assessing two perspectives simultaneously. First, the general contextual effects (GCE) of the neighbourhood on individual health includes all possible mechanisms that can make health and life span among neighbourhood residents more similar than among non-residents, but neglects to define the neighbourhood characteristics behind the similarity. This neighbourhood clustering of health or mortality can be identified with measures of variance and heterogeneity such as intraclass correlation and the median incidence-rate ratio. Second, the associations between specific neighbourhood characteristics (e.g. unemployment rate, mean income level, area deprivation index) and individual health outcomes, known as specific contextual effects (SCE), tend to be evaluated on measures of association such as rate differences and incidence-rate ratios. Both of these effects may be partially attributable to differing population composition among neighbourhoods (i.e. a high proportion of residents with personal characteristics predicting poor health). To the extent that this is true, the origins of the observed neighbourhood effects are unlikely to stem from area-level processes. Nevertheless, even after accounting for numerous individual-level characteristics among residents, several studies report both GCE and SCE with health and mortality outcomes (Meijer et al., 2012).

Previous research has also shown that individual-level socioeconomic mortality patterns are not static across time (Mackenbach et al., 2016). Until recently, the secular trend of declining mortality in high-income countries has been the backdrop in the development of socioeconomic mortality patterns. However, mortality has not declined similarly across the socioeconomic spectrum, leading to narrowing or widening disparities in different national contexts (Mackenbach et al., 2016). These trends manifest on the area level, too. Various studies have explored SCE trends, mostly between neighbourhood socioeconomic deprivation and mortality in urban areas in the UK (Leyland et al., 2007; Norman et al., 2011), Canada (Pampalon et al., 2008), Italy (Marinacci et al., 2004) and Spain (Borrell et al., 2008; Maynou et al., 2016; Rodríguez-Sanz et al., 2016). The results are mixed, indicating increasing, declining and stable neighbourhood disparities even within the same national context (Norman et al., 2011). Some of the studies assessed the extent to which this was attributable to changes in population composition, but were very restricted in terms of measured individual characteristics. In Barcelona, for example, the increasing proportion of international immigrants explained the favourable mortality trend in neighbourhoods with high unemployment, leading to the narrowing of neighbourhood differentials (Rodríguez-Sanz et al., 2016).

There is evidence that socioeconomic disparity in mortality is more pronounced among segments of the working-age population on both the individual (Martikainen et al., 2009) and the area level (Meijer et al., 2012). Despite the decline in the level of mortality attributable to a decline in mortality to ischaemic heart disease and cerebrovascular disease, mostly at older ages, there have been reports of increases in mortality to external causes (accidents, violence, smoking and substance abuse), which are more prevalent among the working-age population and in deprived urban areas (Leyland et al., 2007). In Finland, too, the individual-level disparity in mortality between income groups increased substantially in 1990–2007 due to the rise in alcohol-attributable and external mortality among those with a low income. However, since 2010 the differences have been diminishing again (Tarkiainen et al., 2012, 2017). This implies that external causes of death may also have relevance in area-level mortality disparities in Finland, but also that any long-term changes in disparities are not necessarily monotonic and therefore should be assessed in detailed period analyses.

The unique contributions of this study rests on the following gaps in the literature. Previous studies on trends in mortality concentrated on SCE, and none thus far have explored changes in GCE and therefore have been unable to explore the possible long-term changes in the all-inclusive effect of neighbourhoods on health. Furthermore, it is also unclear whether the changes in socioeconomic disparities occur similarly on the neighbourhood and individual levels. Given the observed increase in individual-level mortality disparity in Finland, a comparison of the disparity trends on both of these levels reveal whether similar or more adverse development is occurring on the neighbourhood level after accounting for the changes in individual-level characteristics. In addition, previous research has often assessed mortality outcomes and have not established whether levels and trends in area-level effects are similar for other, less serious health outcomes such as hospital visits. With a view to narrowing these research gaps in the literature we, therefore, analysed high-quality register data from Finland on the total working-age population living in urban areas, as well as the SCE trends and individual-level associations, and whether these trends were attributable to changes in the composition of neighbourhood populations. We pose the following specific research questions: 1) Did the GCE of neighbourhood residence on mortality and hospitalisation change during the period from 1991 to 2018? 2) Did the neighbourhood SCE of mean income level on these health outcomes change, and were any changes similar to those observed for individual-level income? 3) Are the changes in both GCE and SCE attributable to individual-level characteristics among residents? 4) Are the changes similar for external causes of death and hospitalisations?

## 2 Material & methods

We used individual-level information on the total population of 20–64-year-olds registered as residents in the nine Finnish cities with over 100,000 inhabitants (Helsinki, Espoo, Vantaa, Tampere, Turku, Oulu, Kuopio, Jyväskylä and Lahti) at the end of the baseline years 1990, 1994, 1998, 2002, 2006, 2010 and 2014. We obtained data on the sociodemographic characteristics of the residents, area of residence on the postal-code level, and dates and causes of death originating from the registers of Statistics Finland. Dates of inpatient care episodes in hospitals, including diagnoses from the Finnish Institute for Health and Welfare, were linked to individuals by means of personal identification codes at Statistics Finland (Mähönen et al., 2000). We followed all individuals for four years after each of the seven baseline years for death and the number of years during which they were hospitalised at least once. Deaths and hospitalisations were categorised as external if the underlying cause was alcohol-, injury- or violence-related (International Classification of Diseases 10th revision codes: F10, G312, G4051, G621, G721, I426, K292, K70, K852, K860, O354, P043, Q860, V01–Y89). Postal code areas are suitable as an approximation of a neighbourhood in the included urban areas whereas in rural areas they are too large to be perceived as neighbourhoods. The number of urban post-code areas included in our data decreased from 347 to 343 over the period, and their mean population increased from 2745 to 3500 (interquartile range increased from 807–3881 to 1299–4800).

We estimated random-effects Poisson models for ungrouped data (Loomis et al., 2005) with individuals nested in postal-code areas for each of the seven periods, calculating the median incidence-rate ratio (MIRR) to assess the clustering of the individual level outcomes at the area level, in other words the GCE of the area of residence on the individual hospitalisation or death. MIRR shows the median relative difference in the rate of occurrence of the event when subjects with identical covariates are compared from two random neighbourhoods ordered by rate (Austin et al., 2018). Presenting between-neighbourhood variation using this measure of association enables the comparison of the GCE to the SCE and to the individual-level income associations assessed by the incidence-rate ratios (IRR) in the fixed part of the model.

We explored the period changes in the association between individual and area level income (SCE) and individual hospitalisation or death. The income indicator was the total taxable income of all household members during the baseline year. Because we did not have more detailed information on household structure for all study years we divided the total sum of income by the square root of the number of persons in the household to get an estimate of each individual's available material resources (OECD, 2022a). Only around 1% had zero or missing household income. We then calculated individual-level income quintiles during each of the baseline years using quintile cut-points from the income distribution of all individuals in the data. The area level income quintiles were defined using quintile cut-points defined by the distribution of mean income of the postal-code areas covered by our study resulting in roughly 60 postal-code areas in each quintile. We considered this area income measure to indicate socioeconomic differences between neighbourhoods and being possibly associated with individual level health via mechanisms presented in the introduction. However, given the differing population numbers in the postal-code areas each quintile does not correspond to one fifth of the population. To account for the differing populations in each neighbourhood-income group we calculated ridit scores for individual- and neighbourhood-level income quintiles and included them as covariates in the model, resulting in a relative index of inequality (RII) for both levels (Ernstsen et al., 2012). All the analyses, including the calculation of income-quintile cut-off points, were stratified by sex and baseline year.

We first adjusted the models for age (five-year categories), and then to account for confounding due to differing neighbourhood population composition we adjusted for other individual-level characteristics including education (basic, intermediate, high), taxable household income quintile, housing tenure (owner occupier, renter, other), marital status (never married, married, divorced, widowed), employment status (employed, unemployed, retired, other), immigrant background (both parents born outside Finland, other) and living alone (dwelling alone, other), as described elsewhere (Statistics Finland, 2022a, 2022b, 2022c).

## 3 Results

Table 1 gives the mean ages of the study population aged 20–64 at baseline and the distributions of the individual-level variables for each period used in the analysis. It shows increases from 1991 to 2018 in mean age, general level of education, and proportions of never-married, renters, non-employed, immigrants, those dwelling alone and in the income gap between high- and low-income areas. Table 2 reveals substantial disparities in age-adjusted all-cause and external mortality and hospitalisation rates between income quintiles on the individual level. The disparity between income quintiles on the neighbourhood level is substantially smaller than on the individual level. Mortality and hospitalisation rates declined between the first and the last period in all income quintiles on both levels. We observed a narrowing difference in mortality between the extreme income quintiles on the individual and neighbourhood levels among men, but for hospitalisations the gap increased except for neighbourhood differences in all causes. However, among women the differences increased on both individual and area levels for all outcomes except all-cause hospitalisations, which declined slightly.

**Table 1:** Percentage distributions of individual-level population characteristics at each baseline year, Finnish men and women aged 20-64.

	1990	1994	1998	2002	2006	2010	2014
Sex							
Male	47.6	47.8	48.0	48.2	48.4	48.7	49.0
Female	52.4	52.2	52.0	51.8	51.6	51.3	51.0
Age (mean)							
	39.6	40.1	40.2	40.4	40.7	40.8	40.2
Education							
Basic	34.0	30.0	25.1	21.8	19.4	17.8	16.1
Intermediate	36.8	37.7	39.7	41.4	42.4	41.8	41.9
High	29.2	32.3	35.2	36.8	38.2	40.4	42.0
Marital status							
Never married	33.6	35.4	38.8	41.6	43.5	45.0	47.7
Married	52.9	50.4	46.6	43.8	42.3	41.4	39.4
Divorced	11.1	12.3	12.9	13.1	12.9	12.5	12.0
Widowed	2.3	2.0	1.7	1.5	1.3	1.1	0.9
Tenure							
Owner	67.4	60.3	55.4	51.2	53.5	53.4	51.6
Renter	31.1	36.8	40.9	44.5	41.4	41.5	43.7
Other	1.6	3.0	3.7	4.3	5.1	5.1	4.7
Economic activity							
Employed	79.1	63.5	69.2	72.3	73.9	73.7	72.9
Unemployed	1.5	14.7	10.2	7.5	6.5	6.7	8.5
Retired	9.4	9.6	8.5	8.1	6.7	7.0	5.8
Other	10.0	12.1	12.1	12.1	12.9	12.6	12.7
Dwelling alone							
No	81.7	78.9	77.3	75.7	73.8	73.8	73.8
Yes	18.3	21.1	22.7	24.3	26.2	26.2	26.2
Immigrant background							
No	98.5	97.2	96.3	95.3	93.8	91.6	88.9
Yes	1.5	2.8	3.7	4.7	6.2	8.4	11.1

Gap between high and low area income quintile (2021 euros)	20,651	15,374	18,393	21,315	23,179	24,772	25,265
N	952,493	985,012	1,046,380	1,098,397	1,122,128	1,166,370	1,200,431

**Table 2:** Age-adjusted mortality and hospitalisation rates (per 10,000 person years) attributable to all causes and external causes by individual-income and area-income quintile during the periods 1991–94 (1991) and 2015–18 (2015).

	Men								Women							
	Individual income				Area income				Individual income				Area income			
	Mortality		Hospitalisation		Mortality		Hospitalisation		Mortality		Hospitalisation		Mortality		Hospitalisation	
	1991	2015	1991	2015	1991	2015	1991	2015	1991	2015	1991	2015	1991	2015	1991	2015
**All cause**																
5th quintile (highest)	29	13	549	299	40	21	558	322	16	8	626	317	21	12	626	329
4th quintile	37	19	595	335	49	26	602	352	18	11	652	351	22	14	639	369
3rd quintile	44	26	620	366	56	29	652	379	20	13	667	374	24	15	684	389
2nd quintile	56	41	657	415	59	37	655	396	25	21	673	413	24	18	688	400
1st quintile (lowest)	110	79	764	532	62	38	688	416	41	39	755	523	25	20	731	432
Difference 1–5 quintile	81	66	216	233	21	16	130	95	25	31	129	206	4	8	105	102
N	9531	7161	113,234	88,004	9531	7161	113,234	88,004	4582	3954	133,539	94,562	4582	3954	133,539	94,562
**External**																
5th quintile (highest)	10	4	141	74	14	7	160	93	3	1	94	63	5	3	111	74
4th quintile	12	6	158	89	18	8	184	107	4	2	101	69	5	3	125	86
3rd quintile	14	8	173	103	19	10	204	121	4	3	112	80	6	4	135	94
2nd quintile	19	12	200	138	21	13	203	137	6	4	131	107	6	4	130	103
1st quintile (lowest)	44	31	318	257	21	13	212	148	13	12	222	194	6	5	141	118
Difference 1–5 quintile	34	27	177	183	7	6	52	55	10	11	128	131	1	2	30	44
N	3303	2386	34,689	28,756	3303	2386	34,689	28,756	1112	899	25,481	23,626	1112	899	25,481	23,626

**Fig. 1:**
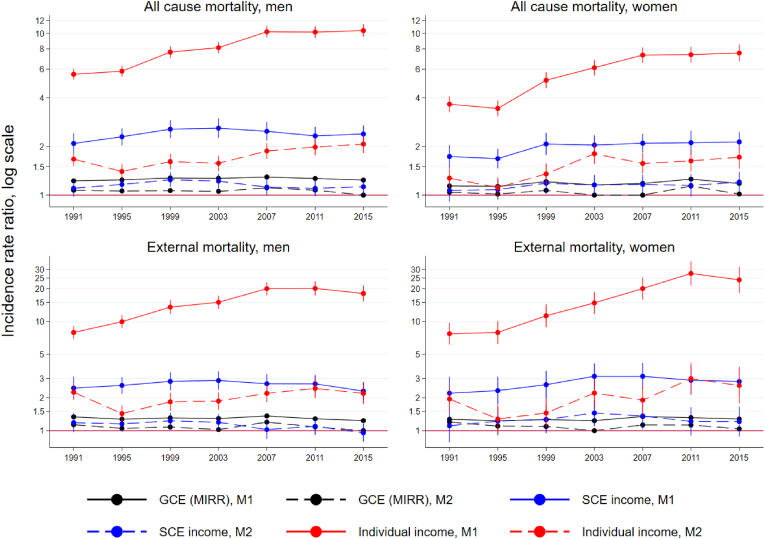
Trends in all-cause and external mortality disparities: median incidence rate ratios (MIRR) for general contextual effect (GCE), relative index of inequality (RII) for area income levels (SCE) and relative index of inequality for individual income in two models adjusted for age (M1, solid line) and all individual characteristics (M2, dotted line), men and women aged 20–64 in 1991–2018.

Fig. 1 shows the relative differences in mortality and hospitalisation between the individual and area-level (SCE) income quintiles summarised as a RII, and the GCE within each study period adjusted for age (continuous line) and other individual-level characteristics (dotted line). When age was accounted for in model 1, the income-related disparity in all-cause mortality was substantially larger on the individual than on the area level, and a clear increasing trend in disparity became visible on the individual level from the mid-1990s, flattening towards the later periods. The upward trend is visible in both sexes, but RII increases more for women, ending up below eight during the last period while for men it settles above ten. The area-level disparity in mortality (SCE) shows a slight initial increase followed by a decline or flattening for both sexes, but the RII of women increases more (to 2.13; 95% CI: 1.85–2.46) and almost reaches the men's level (2.39; 95% CI: 2.12–2.69). GCE measured by the median incidence-rate ratio remains at a low level between 1.23 and 1.30 among men, and around 1.20 among women (see Supplementary Table 1 and 2 for the exact estimates). This means that two randomly chosen individuals ordered by neighbourhood mortality differ by 20–30% in their incidence rate. The changes in GCE across the periods are small.

Accounting for the population composition in model 2 attenuated the disparities markedly, but the changes over time are roughly similar in both models. The individual-level RII remains substantial, but the GCE varies in range from 1.00 to 1.14 and the SCE from 1.10 to 1.25 after these adjustments. The magnitude of the disparity for external causes of death is stronger in model 1, particularly on the individual level. The trends are quite similar to those for all-cause mortality. However, even after adjustment for population composition the decline in RII on both levels towards the last period is clearer for both sexes in model 2, but the disparity is wider among women.

**Fig. 2:**
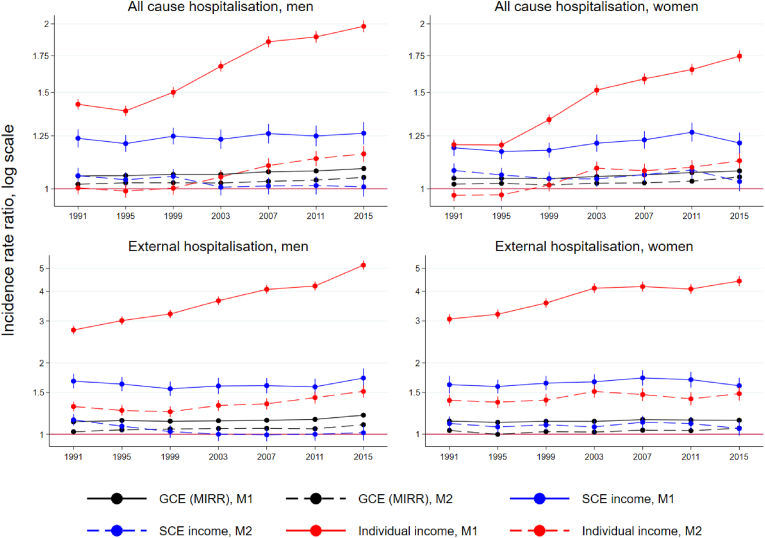
Trends in all-cause and external hospitalisation disparities: median incidence rate ratios (MIRR) for general contextual effect (GCE), relative index of inequality (RII) for area income levels (SCE) and relative index of inequality for individual income in two models adjusted for age (M1, solid line) and all individual characteristics (M2, dotted line), men and women aged 20–64 in 1991–2018.

Fig. 2 shows the trends in hospitalisation outcomes. The magnitude of the disparity in the age-adjusted model is substantially lower for all-cause hospitalisations compared to mortality, but the trends are generally similar. On the individual level, however, the increase continues from the mid-1990s to the last period. The SCE remains unchanged among men, and increases among women close to the men's level at about 1.25. The GCE remains stable at a low level across the periods for both sexes in the range of 1.04–1.09. Adjusting for population composition produced the same result as for mortality: disparity measured in terms of GCE and SCE attenuated to very low levels but the trend remained roughly similar. SCE among men is practically non-existent, and very modest among women, fluctuating between 1.03 and 1.08. The magnitude of the disparities in hospitalisation attributable to external causes is stronger in model 1, but the trends are very similar to those in the all-cause analysis. The area-level disparities attenuated to very low levels following the adjustments to model 2.

For a more detailed picture of the non-linearity of the association between area-level income and our outcomes, and to test whether it had changed from the first to the last period, we ran a Poisson regression model including the interaction of these periods and the area income quintile (Table 3). We observed a clear gradient for age-adjusted all-cause mortality by area income quintile among both men and women during the first period. The disparity between the periods in the lowest income quintile had strengthened, IRR increasing from 1.87 to 2.10 for men and from 1.61 to 2.00 for women (period interaction terms 1.12; 95% CI: 1.00–1.26 and 1.34; 95% CI: 1.15–1.56, respectively; see Supplementary Table 3 for all interaction terms). There was no increase in disparity in the other quintiles, except among women in the second lowest.

**Table 3:** Incidence-rate ratios of all-cause and external mortality and hospitalisation: by area income quintile (SCE) and RII adjusted for age (Model 1) and all individual characteristics (Model 2), men and women aged 20–64 in 1991–94 and 2015-18.

		Men								Women							
		All cause		All cause		External		External		All cause		All cause		External		External	
		Model 1		Model 2		Model 1		Model 2		Model 1		Model 2		Model 1		Model 2	
Period	Mortality	IRR	CI	IRR	CI	IRR	CI	IRR	CI	IRR	CI	IRR	CI	IRR	CI	IRR	CI
1991	5th quintile	1.00		1.00		1.00		1.00		1.00		1.00		1.00		1.00	
1991	4th quintile	1.24	[1.15,1.33]	1.02	[0.95,1.10]	1.32	[1.16,1.49]	1.08	[0.95,1.23]	1.07	[0.97,1.19]	0.94	[0.84,1.04]	1.07	[0.87,1.31]	0.89	[0.73,1.10]
1991	3rd quintile	1.47	[1.38,1.58]	1.03	[0.96,1.11]	1.43	[1.27,1.61]	1.00	[0.89,1.13]	1.23	[1.12,1.35]	0.93	[0.84,1.03]	1.15	[0.95,1.40]	0.80	[0.66,0.98]
1991	2nd quintile	1.72	[1.59,1.85]	1.07	[0.99,1.15]	1.77	[1.56,2.02]	1.08	[0.95,1.23]	1.36	[1.22,1.51]	0.97	[0.87,1.08]	1.60	[1.29,1.98]	1.04	[0.84,1.28]
1991	1st quintile	1.87	[1.72,2.04]	1.04	[0.96,1.14]	1.86	[1.61,2.16]	1.01	[0.87,1.17]	1.49	[1.32,1.68]	0.94	[0.83,1.07]	1.66	[1.29,2.12]	0.91	[0.71,1.18]
1991	RII	2.08	[1.91,2.28]	1.07	[0.98,1.17]	2.05	[1.76,2.38]	1.01	[0.87,1.18]	1.61	[1.42,1.83]	0.97	[0.85,1.10]	1.94	[1.49,2.52]	1.00	[0.77,1.31]
2015	5th quintile	1.00		1.00		1.00		1.00		1.00		1.00		1.00		1.00	
2015 a	4th quintile	1.30	[1.18,1.42]	1.07	[0.97,1.17]	1.29	[1.10,1.53]	1.04	[0.88,1.23]	1.22	[1.08,1.38]	1.06	[0.93,1.19]	1.23	[0.94,1.61]	1.00	[0.77,1.31]
2015 a	3rd quintile	1.49	[1.36,1.63]	1.09	[1.00,1.19]	1.60	[1.36,1.87]	1.12	[0.95,1.31]	1.39	[1.24,1.57]	*1.12*	*[0.99,1.26]*	1.56	[1.21,2.01]	*1.12*	*[0.87,1.45]*
2015 a	2nd quintile	1.86	[1.71,2.03]	1.17	[1.07,1.27]	1.97	[1.69,2.29]	1.15	[0.99,1.35]	*1.63*	*[1.46,1.83]*	*1.20*	*[1.06,1.34]*	1.90	[1.49,2.43]	1.19	[0.93,1.53]
2015 a	1st quintile	*2.10*	*[1.92,2.30]*	1.15	[1.05,1.26]	*2.27*	*[1.94,2.67]*	1.12	[0.95,1.32]	*2.00*	*[1.77,2.25]*	*1.30*	*[1.15,1.47]*	*2.64*	*[2.04,3.41]*	*1.37*	*[1.05,1.78]*
2015 a	RII	*2.39*	*[2.17,2.62]*	1.17	[1.06,1.29]	*2.62*	*[2.22,3.08]*	1.13	[0.96,1.34]	*2.25*	*[1.98,2.55]*	*1.37*	*[1.20,1.56]*	*3.17*	*[2.42,4.15]*	*1.48*	*[1.12,1.96]*
**Hospitalisation**																	
1991	5th quintile	1.00		1.00		1.00		1.00		1.00		1.00		1.00		1.00	
1991	4th quintile	1.09	[1.06,1.11]	1.03	[1.01,1.05]	1.17	[1.13,1.22]	1.06	[1.02,1.10]	1.02	[1.00,1.04]	0.99	[0.97,1.01]	1.16	[1.11,1.22]	1.06	[1.02,1.11]
1991	3rd quintile	1.16	[1.13,1.18]	1.05	[1.03,1.07]	1.30	[1.25,1.34]	1.07	[1.03,1.11]	1.08	[1.06,1.10]	1.01	[0.99,1.03]	1.23	[1.18,1.28]	1.03	[0.99,1.08]
1991	2nd quintile	1.15	[1.12,1.17]	1.02	[1.00,1.04]	1.35	[1.30,1.41]	1.05	[1.01,1.09]	1.06	[1.04,1.08]	0.98	[0.96,1.00]	1.24	[1.18,1.30]	1.00	[0.95,1.05]
1991	1st quintile	1.18	[1.15,1.21]	1.02	[0.99,1.04]	1.41	[1.35,1.47]	1.02	[0.98,1.07]	1.10	[1.07,1.12]	0.99	[0.97,1.01]	1.33	[1.26,1.40]	0.99	[0.94,1.04]
1991	RII	1.19	[1.16,1.22]	1.01	[0.98,1.04]	1.46	[1.40,1.53]	1.01	[0.97,1.06]	1.10	[1.07,1.13]	0.98	[0.96,1.01]	1.32	[1.25,1.39]	0.94	[0.89,0.99]
2015	5th quintile	1.00		1.00		1.00		1.00		1.00		1.00		1.00		1.00	
2015 a	4th quintile	1.06	[1.03,1.09]	1.01	[0.99,1.04]	1.14	[1.09,1.19]	1.02	[0.97,1.07]	*1.10*	*[1.07,1.12]*	*1.07*	*[1.04,1.09]*	1.17	[1.12,1.23]	1.07	[1.01,1.12]
2015 a	3rd quintile	1.16	[1.13,1.18]	1.07	[1.05,1.10]	1.34	[1.28,1.40]	1.12	[1.07,1.17]	*1.17*	*[1.15,1.20]*	*1.12*	*[1.10,1.15]*	1.33	[1.26,1.39]	1.14	[1.09,1.20]
2015 a	2nd quintile	*1.19*	*[1.17,1.22]*	*1.07*	*[1.04,1.09]*	1.49	[1.43,1.55]	1.12	[1.07,1.17]	*1.20*	*[1.17,1.23]*	*1.12*	*[1.10,1.15]*	1.42	[1.36,1.49]	1.13	[1.08,1.19]
2015 a	1st quintile	1.21	[1.18,1.24]	1.04	[1.01,1.06]	1.63	[1.56,1.70]	1.10	[1.05,1.15]	*1.26*	*[1.23,1.29]*	*1.14*	*[1.11,1.17]*	1.67	[1.59,1.76]	1.20	[1.14,1.26]
2015 a	RII	*1.26*	*[1.23,1.29]*	*1.04*	*[1.02,1.07]*	*1.79*	*[1.70,1.87]*	*1.11*	*[1.06,1.17]*	*1.28*	*[1.25,1.32]*	*1.14*	*[1.11,1.17]*	*1.79*	*[1.70,1.88]*	*1.20*	*[1.13,1.26]*

a Estimates for 2015 that differ from 1991 at a 95% confidence level are in italics, exact *P*-values in the Supplementary Table 3.

When we accounted for the sociodemographic composition of the area income quintiles in model 2 the association was non-existent during the first period, but it emerged in the last period, with an excess mortality of 15% and 30% in the lowest income quintiles for men and women, respectively. However, among men the 95% confidence interval of interaction term included 1.00. The development for external causes of death was largely similar to that for all-cause mortality. With regard to hospitalisations, there was no increase in disparities between the extreme neighbourhood-income quintiles among men, and the magnitude was attributable to population composition during the first and last periods. However, the disparities in all-cause and external hospitalisations also increased among women when we accounted for the population composition in model 2.

In general, the disparities in age-adjusted mortality among men increased only in the lowest income areas, although this was attributable to compositional change, whereas among women the widening occurred across all area income quintiles even after accounting for compositional change.

## 4 Discussion

### 4.1 Principal findings

According to the analysis, the general relevance of the neighbourhood context to mortality and hospitalisation among residents (GCE) was stable across time, the risk difference between two individuals in randomly chosen areas being around 20%. The disparities were substantial according to the mean neighbourhood income level (SCE), and increased over time particularly among women. However, these socio-spatial health disparities seemed generally to originate from the differing composition of neighbourhood populations, albeit the trends were relatively unaffected by compositional factors. Among women in particular, the increases were not fully attributable to the sociodemographic composition of the neighbourhoods. The SCE trends in area income did not directly follow the individual-level associations, the increase in health disparity being much stronger on the individual level than on the area level, and in some periods the trends were in opposing directions.

### 4.2 Interpretation of the findings

Both the magnitude and relative stability of GCE and the fact that area-income SCE does not follow the individual-level trend of disparity very closely implies that the neighbourhood context is most likely not very relevant for the health and life-span of 20–64-year-old Finns living in urban areas. This may be partially attributable to the level of socioeconomic residential segregation. Although relatively low in international terms, segregation in Finnish cities has increased slightly during the 2000s, which is visible in the gap between high and low income areas in Table 1, (Lönnqvist et al., 2014; Saikkonen et al., 2018). Low levels of segregation hinder the development of neighbourhood processes that could be hazardous to health over and above individual-level processes. Our findings indicate that such processes are unlikely to be major contributing factors to mortality and hospitalisation. The negative development of all-cause and particularly external mortality among individuals on a low income does not seem to originate from economically deprived neighbourhoods, as population segments on a low income and with poor health are more evenly spread among different neighbourhoods.

Macro-level processes such as economic downturns may also affect the observed area-level association between income and mortality. Our analysis covers a period when two economic crises hit Finland. The depression in the early 1990s saw the unemployment rate peaking at 16% in 1994, which hit low-income neighbourhoods particularly hard (OECD, 2022b). The recession after the financial crisis of 2008 and the subsequent rise in unemployment had a more limited impact than in other European countries (OECD, 2022b). Although, the evidence is not clear-cut, many studies reveal widening health inequalities following economic recessions (Benach et al., 2022; Heggebø et al., 2019). However, evidence of the impact of recessions on neighbourhood-level inequality in mortality is scarce (Benach et al., 2022). One study covering nine European urban areas reported no increase in intra-urban inequalities after the 2008 recession (Palència et al., 2020), but another demonstrated increasing inequality following the same crisis in Barcelona (Maynou et al., 2016). Our results are in line with findings from previous studies in that area-level inequalities did not change following these recessions. On the other hand, we offer contrasting individual-level evidence in that inequalities increased as the economy expanded, and mainly narrowed or stabilised after recessions (from 1991–94 to 1995–98 and 2007–10 to 2011–14). This observation reflects the findings from a previous study concentrating on the recession in Finland during the early 1990s (Valkonen et al., 2000).

We also observed an increase in relative socioeconomic disparities in mortality on the individual level, which has been attributed in part to declining levels of mortality while absolute disparities have been narrowing in several European countries (Mackenbach et al., 2016). This development also seems to be prevalent among men on the area level: most absolute disparities narrowed between the first and last periods whereas relative disparity increased slightly or remained stable. However, the increase in area-level income disparities in mortality and hospitalisation among women was both absolute and relative, and was not attributable to the changing social composition of neighbourhoods. Similar individual-level increases in social disparity affecting mortality among women has been observed in Sweden, Norway and Finland, partially attributable to the widening disparity in smoking- and alcohol-related causes of death (Mackenbach et al., 2016). We suggest that the similar concentration of smoking and alcohol consumption among women in poor areas may produce increasing relative area-level health differentials given the observed increase in mortality attributable to external causes including alcohol-attributable deaths and a rather low absolute level of external mortality among women. However, we have no direct basis on which to evaluate this possibility.

### 4.3 Methodological considerations

On the one hand, area-level processes and mechanisms producing health disparities may not coincide with the administrative postal-code neighbourhood definitions used in this study. This may dilute the true area-level effects and bias our estimates downwards. However, as reported in an earlier study, changing neighbourhood definitions in the Finnish context did not have a substantial impact on disparities in neighbourhood mortality (Tarkiainen et al., 2010). On the other hand, it is unlikely that we were able to account for all relevant individual-level characteristics in our analysis. Hence, failing to adjust for possible sources of confounding may have biased our results upwards and do not allow for firm causal inference.

When using inpatient hospitalisation as a measure of population health it should be noted that the observed decline over time does not only reflect an improvement in population health. Recent reforms and technological improvements in the Finnish healthcare system aimed at directing treatment towards outpatient care (Vuorenkoski, 2008), which is partially reflected in declining hospitalisation rates. However, this is unlikely to have biased our analysis: we have no reason to assume that these changes are dependent on the neighbourhood of residence in that specialised healthcare in hospitals is organised on the municipal or city level.

## 5 Conclusion

The overall relevance of the neighbourhood context on mortality and hospitalisation is stable across time and is largely attributable to population composition. However, there were substantial specific area-level income disparities between neighbourhoods, especially in external causes of death, which even after adjustments widened over time, particularly among women. All area-level income associations were much smaller than corresponding individual-level associations, implying that most processes behind health inequalities operate on other than the neighbourhood level. This should be accounted for in measures introduced to combat inequalities.

## Financial disclosure

None of the authors have any financial disclosures to make.

## Author contribution

LT and PM contributed to the conception and design of the study. PM acquired the data. LT analysed the data and drafted the manuscript. LT and PM contributed substantially to designing the final analyses and revised critically the drafts and the final manuscript for important intellectual content and approved the version published. LT is the guarantor.

## Ethical statement

The register linkages were approved by ethical committees of Statistics Finland and FinData (permissions TK-54-1490-18 and THL/2180/14.02.00/2020).

## Declaration of competing interest

None of the authors have any conflicts of interest to declare.

## Data Availability

Restrictions apply to the availability of the data that support the findings of this study but are available from Statistics Finland and the Finnish Institute for Health and Welfare.
