# Supplementary material for: Long-term trends in urban-neighbourhood inequalities in cause-specific mortality and hospitalisation – multilevel analyses among individuals nested in Finnish post-code areas, 1991–2018
Source: SSM Popul Health. 2022 Dec 17;21:101323. doi: 10.1016/j.ssmph.2022.101323 (PMC9798161; doi:10.1016/j.ssmph.2022.101323)
Supplement: Multimedia component 1 [file mmc1.pdf]

Supplementary Table 1. Estimates of disparities in all-cause and external mortality. The measures include: median incidence rate ratios (MIRR) for general contextual effect (GCE), relative index of inequality (RII) for area income level (SCE) and for individual income in two models adjusted for age (M1) and all individual characteristics (M2); women aged 20-64 in 1991-2018

Period	GCE M1 MIRR	GCE M2 MIRR	Individual income M1 RII	95% CI low	up	Individual income M2 RII	95% CI low	up	SCE M1 RII	95% CI low	up	SCE M2 RII	95% CI low	up
All cause mortality women, 20-64														
1991	1.14	1.04	3.65	3.26	4.08	1.27	1.11	1.46	1.73	1.47	2.04	1.07	0.91	1.26
1995	1.13	1.02	3.43	3.07	3.84	1.11	0.97	1.28	1.68	1.46	1.93	1.08	0.94	1.25
1999	1.21	1.07	5.13	4.58	5.75	1.35	1.17	1.57	2.07	1.77	2.42	1.19	1.02	1.38
2003	1.16	1.00	6.14	5.50	6.85	1.80	1.56	2.09	2.04	1.78	2.34	1.15	1.00	1.33
2007	1.18	1.00	7.34	6.57	8.19	1.57	1.35	1.82	2.09	1.83	2.39	1.17	1.02	1.33
2011	1.26	1.14	7.38	6.60	8.26	1.63	1.40	1.90	2.11	1.78	2.50	1.15	0.98	1.35
2015	1.18	1.02	7.56	6.71	8.51	1.72	1.45	2.03	2.13	1.85	2.46	1.20	1.04	1.40
External mortality women, 20-64														
1991	1.27	1.20	7.74	6.14	9.76	1.95	1.48	2.57	2.21	1.56	3.11	1.11	0.78	1.57
1995	1.23	1.10	7.95	6.25	10.11	1.28	0.95	1.73	2.33	1.74	3.12	1.22	0.91	1.64
1999	1.26	1.09	11.30	8.91	14.34	1.45	1.07	1.98	2.64	1.98	3.52	1.27	0.95	1.69
2003	1.23	1.00	14.82	11.77	18.67	2.21	1.64	2.98	3.15	2.41	4.13	1.45	1.09	1.93
2007	1.35	1.13	20.05	15.87	25.34	1.90	1.40	2.59	3.15	2.37	4.18	1.36	1.03	1.80
2011	1.32	1.13	27.60	21.33	35.71	3.00	2.13	4.21	2.90	2.14	3.93	1.22	0.90	1.64
2015	1.28	1.04	24.09	18.26	31.79	2.59	1.79	3.77	2.82	2.08	3.83	1.21	0.89	1.66
All cause hospitalisations women, 20-64														
1991	1.05	1.02	1.20	1.18	1.23	0.97	0.95	1.00	1.19	1.15	1.23	1.08	1.05	1.11
1995	1.04	1.02	1.20	1.18	1.23	0.97	0.95	1.00	1.17	1.13	1.21	1.06	1.03	1.09
1999	1.04	1.02	1.34	1.31	1.37	1.02	0.99	1.04	1.18	1.14	1.21	1.04	1.01	1.07
2003	1.05	1.02	1.51	1.48	1.55	1.09	1.06	1.12	1.21	1.17	1.26	1.04	1.01	1.08
2007	1.06	1.03	1.59	1.55	1.62	1.08	1.05	1.11	1.23	1.18	1.27	1.06	1.03	1.10
2011	1.07	1.03	1.65	1.61	1.69	1.09	1.06	1.13	1.27	1.22	1.32	1.08	1.05	1.12
2015	1.08	1.05	1.75	1.71	1.79	1.13	1.09	1.16	1.21	1.16	1.27	1.03	0.99	1.07
External hospitalisations women, 20-64														
1991	1.14	1.04	3.05	2.91	3.20	1.39	1.31	1.47	1.62	1.49	1.76	1.11	1.04	1.19
1995	1.12	1.00	3.20	3.06	3.35	1.37	1.29	1.45	1.59	1.49	1.70	1.07	1.01	1.14
1999	1.13	1.03	3.57	3.41	3.74	1.39	1.31	1.48	1.64	1.53	1.76	1.10	1.03	1.16
2003	1.13	1.02	4.12	3.93	4.32	1.52	1.43	1.61	1.66	1.55	1.79	1.07	1.01	1.14
2007	1.15	1.04	4.19	3.99	4.39	1.47	1.38	1.56	1.73	1.60	1.86	1.12	1.06	1.19
2011	1.15	1.04	4.09	3.90	4.29	1.41	1.32	1.50	1.70	1.58	1.83	1.11	1.04	1.18
2015	1.15	1.06	4.42	4.20	4.64	1.48	1.39	1.58	1.60	1.48	1.73	1.06	0.99	1.13

Supplementary Table 2. Estimates of disparities in all-cause and external mortality. The measures include: median incidence rate ratios (MIRR) for general contextual effect (GCE), relative index of inequality (RII) for area income level (SCE) and for individual income in two models adjusted for age (M1) and all individual characteristics (M2); men aged 20-64 in 1991-2018

Period	GCE M1 MIRR	GCE M2 MIRR	Individual income M1 RII	95% CI low	up	Individual income M2 RII	95% CI low	up	SCE M1 RII	95% CI low	up	SCE M2 RII	95% CI low	up
All cause mortality men, 20-64														
1991	1.23	1.07	5.59	5.17	6.04	1.67	1.52	1.84	2.09	1.81	2.41	1.10	0.98	1.24
1995	1.24	1.06	5.84	5.39	6.32	1.40	1.26	1.55	2.29	2.04	2.59	1.17	1.05	1.29
1999	1.28	1.07	7.65	7.05	8.31	1.61	1.44	1.80	2.56	2.25	2.90	1.25	1.12	1.38
2003	1.27	1.06	8.15	7.52	8.83	1.57	1.41	1.75	2.59	2.26	2.98	1.22	1.10	1.36
2007	1.30	1.11	10.23	9.43	11.09	1.88	1.68	2.10	2.48	2.17	2.83	1.12	1.00	1.26
2011	1.27	1.07	10.19	9.37	11.07	1.98	1.76	2.23	2.32	2.04	2.64	1.10	0.99	1.23
2015	1.24	1.00	10.41	9.53	11.37	2.07	1.82	2.35	2.39	2.12	2.69	1.13	1.01	1.26
External mortality men, 20-64														
1991	1.34	1.13	7.95	6.94	9.10	2.25	1.92	2.63	2.46	1.93	3.12	1.18	0.97	1.45
1995	1.27	1.05	9.97	8.68	11.44	1.43	1.20	1.71	2.60	2.17	3.10	1.15	0.98	1.36
1999	1.31	1.08	13.57	11.74	15.68	1.84	1.52	2.22	2.83	2.34	3.42	1.23	1.04	1.46
2003	1.29	1.02	15.02	13.06	17.27	1.87	1.55	2.25	2.88	2.37	3.50	1.19	1.01	1.41
2007	1.36	1.20	20.03	17.41	23.05	2.20	1.82	2.66	2.69	2.19	3.31	1.02	0.84	1.24
2011	1.29	1.08	20.12	17.33	23.37	2.44	1.99	2.99	2.69	2.24	3.23	1.10	0.92	1.31
2015	1.24	1.00	18.09	15.35	21.31	2.20	1.75	2.77	2.30	1.90	2.79	0.96	0.79	1.16
All cause hospitalisations men, 20-64														
1991	1.06	1.02	1.43	1.40	1.46	1.00	0.98	1.03	1.24	1.19	1.28	1.06	1.02	1.09
1995	1.06	1.03	1.39	1.36	1.42	0.99	0.96	1.02	1.21	1.17	1.25	1.04	1.01	1.07
1999	1.06	1.03	1.50	1.47	1.53	1.00	0.97	1.03	1.25	1.20	1.29	1.05	1.02	1.09
2003	1.06	1.02	1.67	1.64	1.71	1.05	1.02	1.08	1.23	1.18	1.28	1.01	0.97	1.04
2007	1.07	1.03	1.86	1.81	1.90	1.10	1.07	1.14	1.26	1.21	1.31	1.01	0.98	1.05
2011	1.08	1.04	1.89	1.85	1.94	1.14	1.10	1.17	1.25	1.20	1.30	1.01	0.98	1.05
2015	1.09	1.05	1.98	1.93	2.03	1.16	1.12	1.20	1.26	1.21	1.32	1.01	0.97	1.05
External hospitalisations men, 20-64														
1991	1.13	1.03	2.75	2.64	2.86	1.31	1.25	1.37	1.67	1.56	1.80	1.15	1.09	1.22
1995	1.14	1.05	3.01	2.89	3.13	1.26	1.20	1.33	1.63	1.52	1.74	1.08	1.02	1.14
1999	1.14	1.05	3.21	3.08	3.34	1.25	1.18	1.31	1.56	1.45	1.67	1.03	0.97	1.09
2003	1.14	1.06	3.65	3.50	3.80	1.32	1.26	1.39	1.60	1.48	1.73	1.00	0.94	1.07
2007	1.15	1.06	4.08	3.91	4.25	1.34	1.27	1.42	1.60	1.49	1.73	0.99	0.93	1.06
2011	1.16	1.06	4.21	4.03	4.40	1.43	1.35	1.51	1.59	1.47	1.72	1.00	0.94	1.07
2015	1.20	1.10	5.15	4.92	5.39	1.52	1.43	1.61	1.73	1.57	1.89	1.02	0.94	1.10

Supplementary Table 3. Interaction terms of changes in incidence-rate ratios from 1991-94 to 2015-18: all-cause and external mortality and hospitalisation by area income quintile (SCE), RII adjusted for age (Model 1) and all individual characteristics (Model 2); men and women aged 20-64

**Men**	All cause				All cause				External				External			
	Model 1	95% CI			Model 2	95% CI			Model 1	95% CI			Model 2	95% CI		
**Mortality**	IRR	low	up	p-value	IRR	low	up	p-value	IRR	low	up	p-value	IRR	low	up	p-value
5th quintile	1				1				1				1			
4th quintile	1.05	0.93	1.18	0.43	1.04	0.93	1.17	0.49	0.98	0.80	1.21	0.86	0.96	0.78	1.18	0.70
3rd quintile	1.01	0.90	1.13	0.87	1.06	0.95	1.18	0.33	1.12	0.92	1.36	0.26	1.12	0.92	1.36	0.25
2nd quintile	1.09	0.98	1.21	0.13	1.09	0.98	1.22	0.10	1.11	0.92	1.34	0.27	1.07	0.88	1.29	0.50
1st quintile	1.12	1.00	1.26	0.04	1.10	0.98	1.23	0.09	1.22	1.00	1.48	0.05	1.11	0.91	1.35	0.31
RII	1.15	1.02	1.28	0.02	1.09	0.98	1.23	0.12	1.28	1.06	1.55	0.01	1.12	0.92	1.36	0.26
**Hospitalisation**																
5th quintile	1				1				1				1			
4th quintile	0.98	0.95	1.01	0.15	0.98	0.95	1.01	0.27	0.97	0.92	1.03	0.35	0.97	0.91	1.03	0.27
3rd quintile	1.00	0.97	1.03	0.96	1.02	0.99	1.05	0.17	1.04	0.98	1.09	0.23	1.05	0.99	1.11	0.12
2nd quintile	1.04	1.01	1.07	0.01	1.05	1.02	1.08	0.00	1.10	1.04	1.16	0.00	1.07	1.01	1.13	0.02
1st quintile	1.03	1.00	1.06	0.08	1.02	0.99	1.05	0.20	1.16	1.09	1.22	0.00	1.08	1.02	1.14	0.01
RII	1.06	1.02	1.09	0.00	1.04	1.00	1.07	0.03	1.22	1.15	1.29	0.00	1.10	1.04	1.16	0.00

**Women**	All cause				All cause				External				External			
	Model 1	95% CI			Model 2	95% CI			Model 1	95% CI			Model 2	95% CI		
**Mortality**	IRR	low	up	p-value	IRR	low	up	p-value	IRR	low	up	p-value	IRR	low	up	p-value
5th quintile	1				1				1				1			
4th quintile	1.13	0.97	1.33	0.12	1.13	0.96	1.32	0.14	1.15	0.82	1.61	0.41	1.12	0.80	1.57	0.50
3rd quintile	1.14	0.98	1.31	0.09	1.20	1.04	1.39	0.01	1.36	0.99	1.85	0.06	1.39	1.02	1.90	0.04
2nd quintile	1.20	1.04	1.39	0.01	1.24	1.07	1.43	0.00	1.19	0.88	1.61	0.26	1.15	0.85	1.56	0.36
1st quintile	1.34	1.15	1.56	0.00	1.37	1.18	1.60	0.00	1.59	1.16	2.19	0.00	1.50	1.09	2.06	0.01
RII	1.40	1.19	1.63	0.00	1.41	1.21	1.65	0.00	1.63	1.18	2.26	0.00	1.48	1.07	2.05	0.02
**Hospitalisation**																
5th quintile	1				1				1				1			
4th quintile	1.07	1.04	1.10	0.00	1.08	1.04	1.11	0.00	1.01	0.94	1.08	0.82	1.00	0.94	1.07	0.97
3rd quintile	1.09	1.06	1.12	0.00	1.11	1.08	1.14	0.00	1.08	1.01	1.14	0.02	1.10	1.04	1.17	0.00
2nd quintile	1.13	1.10	1.17	0.00	1.14	1.11	1.17	0.00	1.15	1.08	1.22	0.00	1.13	1.06	1.20	0.00
1st quintile	1.15	1.12	1.18	0.00	1.16	1.12	1.19	0.00	1.26	1.18	1.34	0.00	1.22	1.14	1.30	0.00
RII	1.17	1.13	1.20	0.00	1.17	1.13	1.20	0.00	1.35	1.27	1.44	0.00	1.27	1.19	1.35	0.00
